# Spinal Muscular Atrophy Therapeutics: Where do we Stand?

**DOI:** 10.1007/s13311-015-0337-y

**Published:** 2015-01-29

**Authors:** Constantin d’Ydewalle, Charlotte J. Sumner

**Affiliations:** 1 Department of Neurology, Johns Hopkins University School of Medicine, 855 North Wolfe St., Baltimore, MD 21205 USA; 2 Department of Neuroscience, Johns Hopkins University School of Medicine, 855 North Wolfe St., Baltimore, MD 21205 USA

**Keywords:** Spinal muscular atrophy, Survival motor neuron, Gene activation, Splicing modulation, Gene therapy

## Abstract

Spinal muscular atrophy (SMA) is an inherited neuromuscular disorder pathologically characterized by the degeneration of motor neurons in the spinal cord and muscle atrophy. Motor neuron loss often results in severe muscle weakness causing affected infants to die before reaching 2 years of age. Patients with milder forms of SMA exhibit slowly progressive muscle weakness over many years. SMA is caused by the loss of *SMN1* and the retention of at least 1 copy of a highly homologous *SMN2*. An alternative splicing event in the pre-mRNA arising from *SMN2* results in the production of low levels of functional SMN protein. To date, there are no effective treatments available to treat patients with SMA. However, over the last 2 decades, the development of SMA mouse models and the identification of therapeutic targets have resulted in a promising drug pipeline for SMA. Here, we highlight some of the therapeutic strategies that have been developed to activate *SMN2* expression, modulate splicing of the *SMN2* pre-mRNA, or replace *SMN1* by gene therapy. After 2 decades of translational research, we now stand within reach of a treatment for SMA.

**Electronic supplementary material:**

The online version of this article (doi:10.1007/s13311-015-0337-y) contains supplementary material, which is available to authorized users.

## Introduction

Spinal muscular atrophy (SMA) is the most common autosomal recessive cause of infant mortality, and was first described by Werdnig and Hoffmann in the early 1890s [1, 2]. SMA affects approximately 1 in 6000–1 in 10,000 live births, and the carrier frequency is estimated to be 1 in 40–1 in 60 [3, 4]. The classical pathological hallmark of SMA is the loss of motor neurons in the anterior horn of the spinal cord. Clinically, patients with SMA are classified into 1 of 5 types based on age of onset and the inability to achieve motor milestones [5, 6]. Type 0 SMA is the most severe form, with onset at neonatal stages and sometimes reduced movement *in utero* [6]. Patients with SMA type 0 can never sit and, if untreated, they do not survive beyond the first months after birth [6]. SMA type 1 is the most common form of the disease, with a clinical onset usually before the age of 6 months. Affected infants never acquire the ability to sit unsupported and often have no head control owing to severe hypotonia and symmetrical paralysis [5, 6]. Patients with SMA type 2 are characterized by an age of onset ranging from 7 to 18 months. While these infants can sit unsupported, they are unable to walk independently [5, 6]. In both types of SMA, the diaphragm muscle is usually spared while intercostal muscles are severely affected. This results in paradoxical breathing; hence, the cause of death in patients with both type 1 and type 2 SMA is usually respiratory failure or complications of the respiratory tract [5, 6]. Prior to recent decades, most patients with SMA type 1 did not survive beyond the first 2 years of life, while patients with type 2 SMA survived into adulthood. [5, 6]. With increased use of respiratory and nutritional support in recent decades, the average survival of patients with SMA type 1 has increased [7, 8]. However, these interventions do not improve muscle strength. Patients with SMA type 3 develop symptoms after the age of 2 years. They usually achieve all major motor milestones, although many need wheelchair assistance later in childhood or adulthood. Patients with SMA type 4 usually have an onset in the second or third decade of life. Muscle weakness is mild to moderate, and they generally have no respiratory problems [5, 6].

**Fig. 1:**
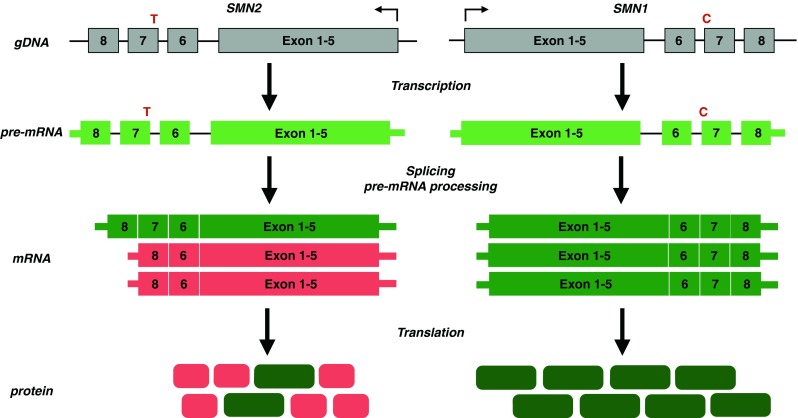
Genetics of spinal muscular atrophy (SMA). SMA is caused by mutation of *SMN1* and reduced survival motor neuron (SMN) protein levels. All patients retain at least 1 copy of the highly homologous *SMN2. SMN2* harbors a translational silent C > T substitution in a splice enhancer sequence of exon 7, resulting in exon 7 skipping at the mRNA level. The alternatively spliced SMN2 mRNA encodes a truncated, highly unstable, nonfunctional protein. A small fraction of SMN2 transcripts contain exon 7 that encode a full-length, functional SMN protein

A century after the first description of SMA, the underlying genetic defect was identified [9]. Genetic linkage analyses and subsequent positional cloning in patients with SMA identified a disease-associated 140-kb region that contained the duplicated *SMN* on chromosome 5q13 [9]. In patients with SMA, the telomeric copy of *SMN* (*SMN* ^*T*^) on 5q13 is lacking usually owing to large deletion mutations or, rarely, to point mutations that disrupt survival motor neuron (SMN) function [9]. The highly homologous centromeric copy *SMN* (*SMN* ^*C*^), located on the same chromosome, contains a critical translational silent C > T substitution in an exonic enhancer at codon 280 in exon 7 (6 base pairs downstream from the 5’ end of exon 7), resulting in an alternatively spliced, truncated and nonfunctional SMN protein (Fig. 1) [9, 10]. Currently, *SMN* ^*T*^ is referred to as *SMN1*, while *SMN* ^*C*^ is called *SMN2*. Humans harbor at least 1 copy of *SMN2*. Soon after the discovery of the disease-causing gene, it became clear that *SMN2* copy numbers inversely correlate with disease severity in most cases [11].

**Fig. 2:**
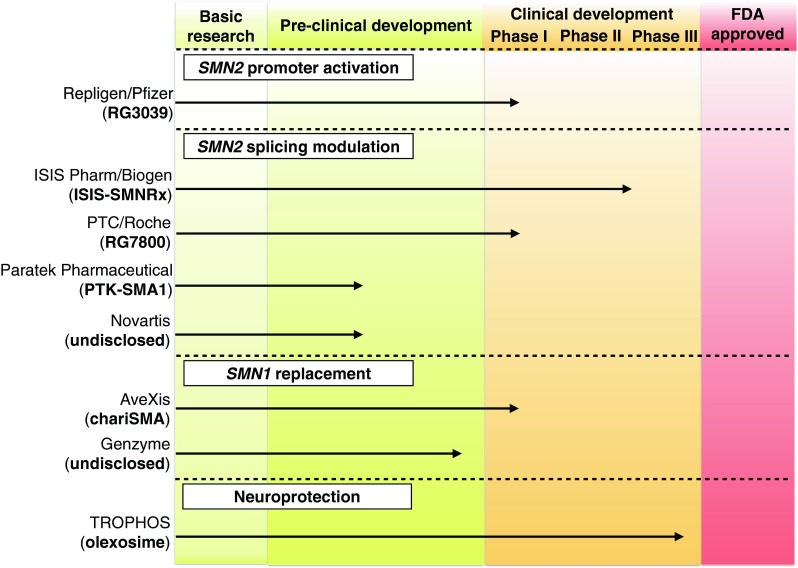
Pipeline of spinal muscular atrophy drugs. The status of 1) *SMN2* promoter activating drugs, 2) *SMN2* splicing modulating drugs, and 3) *SMN1*-replacing strategies that are in advanced stages of development and described in this review. FDA = US Food and Drug Administration

Over the last 2 decades several repurposed drugs with neuroprotective effects have been tested in clinical trials in patients with SMA (reviewed in [12]). Unfortunately, their effectiveness in slowing disease progression was limited. In 2011, the US Food and Drug Administration (FDA) approved, for the first time, a phase I clinical trial of a drug developed specifically for the treatment of SMA (‘RG3039’; Repligen/Pfizer). Since then, other promising new therapeutic candidates have been validated in preclinical models and are now moving to clinical trials in human patients (Fig. 1). In this review, we aim to highlight those promising SMA treatments that are currently in advanced stages of development (summarized in Fig. 2).

## SMN

The SMN protein is a 294-amino acid polypeptide with a predicted size of 38 kDa. It is highly conserved across species and is expressed ubiquitously, raising the question of why motor neurons are specifically vulnerable to SMN deficiency. Both full-length and truncated *SMN* mRNA show similar half-lives in primary fibroblasts, suggesting that mRNA stability does not contribute to differences in SMN protein levels [13]. Levels of functional full-length SMN protein that self-associate into the SMN complex inversely correlate with disease severity [9, 10, 14, 15]. In contrast, truncated SMN protein arising from *SMN2* is unstable and is less efficient in self-associating causing reduced levels of functional SMN complex [15–17]. Thus, *SMN2* fails to compensate for the loss of *SMN1*, resulting in the development of SMA.

SMN localizes both to the cytoplasm and to very distinct structures in the nucleus called “gems”, the function(s) of which are still under investigation [18]. SMN interacts with a wide variety of known RNA binding proteins such as small nuclear ribonucleic particles containing small nuclear RNAs (snRNAs) and small nuclear ribonucleoproteins, as well as other RNA binding proteins [18–20]. Several studies from the Dreyfuss laboratory [21–24] have indicated that SMN plays a crucial role in specific small nuclear ribonucleoprotein biogenesis and spliceosome assembly, and hence in pre-mRNA splicing (for a detailed review on the role of SMN in spliceosome assembly, see [25]). In line with these findings, SMN deficiency causes tissue-specific perturbations in snRNA levels and widespread defects in splicing [24, 26, 27]. A search for specific genes showing perturbed splicing and hence decreased expression in a *Drosophila* model for SMN deficiency identified Stasimon, a protein required for proper motor circuit function [28]. Deep RNA sequencing of microdissected motor neurons and white matter from SMA mice similarly identified mis-spliced genes that are critical for synaptogenesis both in spinal cord and at neuromuscular junctions [29]. The identification of specific genes whose processing is perturbed by SMN deficiency might provide additional information about SMA pathology. Additionally, these genes might represent disease modifiers and thus non-SMN targets for potential new therapeutic strategies.

Some reports have demonstrated that SMN also localizes to cytoplasmic or neuritic granules in neurons suggesting that SMN might also play a neuron-specific role in mRNA transport or local mRNA processing [30–33]. A recent report also indicated that reduced levels of SMN are associated with an increase in microRNA-183 expression, causing downregulation of mammalian target of rapamycin and, eventually, reduced global translation in neurons [34]. These observations indicate that the mammalian target of rapamycin pathway and specific microRNAs might contribute to SMA pathology. Whether SMN has other functions (in neurons and/or in other cell types) is still under investigation.

## SMA Mouse Models

Critical to understanding the pathogenic mechanism underlying SMA, as well as to the development of (new) therapeutic strategies, is the generation of animal models that recapitulate human disease as closely as possible.

In contrast to humans, mice only harbor one *Smn gene*. In 1997, Schrank et al. [35] reported that homozygous deletion of *Smn* causes massive cell death during early embryonic development. This observation emphasizes that some SMN is required for survival of all cells. Embryonic lethality of *Smn* ^–/–^ mice was rescued by inserting 1 (founder line 89) or 8 (founder line 566) copies of the human *SMN2* [36]. At birth, *Smn* ^–/–^/*SMN2* ^+/+^ mice appear normal, irrespective of the number of *SMN2* copies [36]. However, low-copy animals quickly deteriorate with tremor in the hind limbs, reduced righting reflex, severe muscle weakness, and loss of body weight followed by death at the age of 6 days [36]. Interestingly, high-copy *Smn* ^–/–^/*SMN2* ^+/+^ mice do not show any obvious phenotype and live a normal life, consistent with the inverse correlation of disease severity and *SMN2* copy numbers in patients with SMA [36]. Like in humans, most transcripts arising from *SMN2* lack exon 7, and *Smn* ^–/–^/*SMN2* ^+/+^ mice have 10- to 20-fold less SMN protein levels compared with their control littermates [36].

Subsequently, a transgene harboring a 3.4-kb portion of the human *SMN* promoter followed by a truncated *SMN2* cDNA (*SMNΔ7*) clone was introduced into the severe SMA mouse model [37]. The resulting *Smn* ^–/–^/*SMN2* ^+/+^/*SMNΔ7* ^+/+^ (“Delta7”) mice show a prolonged survival (with a mean survival of 13 days) compared with the severe *Smn* ^–/–^/*SMN2* ^+/+^ mice [37].

In parallel work, another mouse model with a SMA-like phenotype was generated [38]. This other widely used model is referred to as the “Taiwanese” SMA mouse model. Homozygous deletion of mouse *Smn* also caused embryonic lethality in this study, confirming that SMN is required in early embryonic stages [38]. Transgene insertion of a 115-kb human genomic region containing *SMN2* and (parts of) neighboring genes rescued embryonic lethality [38]. The resulting SMA-like mice were categorized into 3 groups based on the severity of the phenotype. Type 1 SMA mice displayed body weight loss, did not develop furry hair, and died by the age of 10 days [38]. Mice with intermediate severity (type 2) showed body weight loss and poor activity, and died between 2 weeks and 4 weeks of age [38]. Mice that survived and bred normally but had shorter tails were classified as type 3 [38]. This mouse line has subsequently been bred at The Jackson Laboratory, resulting in a more homogenous phenotype. Other mouse models with modifications of the aforementioned SMA backgrounds, as well as conditional mouse models of SMA, have been reported. Development of mouse models of mild SMA have been challenging (for a review of SMA mouse models, see [39]).

Severe SMA mouse models have been studied extensively to determine the pathological basis of weakness and reduced survival. Interestingly, although SMA mice do exhibit some motor neuron loss at end-stages of the disease, it varies depending on spinal cord region and, overall, may not be sufficient to account for the magnitude of weakness [40, 41]. Similarly, denervation of muscle is restricted to few muscle groups [42]. This has indicated that motor neuron dysfunction rather than death drives clinical manifestations in early stages of disease. Indeed, it has been demonstrated that neuromuscular junctions [41, 43–47], as well as synaptic inputs onto motor neurons in the spinal cord [40, 45], are structurally and functionally abnormal. These functional changes are associated with the presence of widespread hypotrophic muscle fibers consistent with a lack of myofiber maturation [44, 47].

## Is SMA a Neurodevelopmental or a Neurodegenerative Disorder, or Both?

**Fig. 3:**
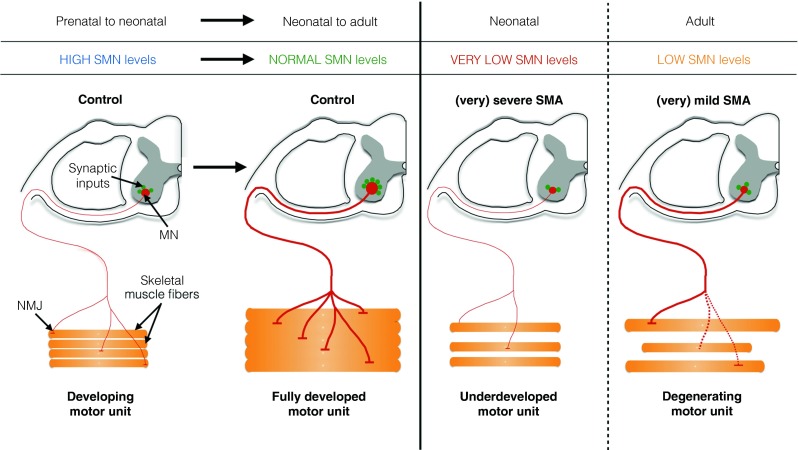
Survival motor neuron (SMN) may be crucial for normal development and postnatal maintenance of the motor unit. In prenatal and perinatal stages, terminally differentiated motor neurons (MN) in the spinal cord express high levels of SMN. At these stages, developing motor axons are still small in diameter, but reach their target muscle correctly. The immature synaptic inputs in the spinal cord, as well as immature neuromuscular junctions (NMJs), are developing in neonatal stages. Once the motor unit is fully developed, SMN levels drop to normal levels. These levels are sufficient to maintain motor unit function and respond to nerve injury. In severe forms of spinal muscular atrophy (SMA; type 0 and 1), very low levels of SMN cause incomplete maturation of the motor unit. Classical features include incomplete development of synaptic inputs of motor neurons in the spinal cord and partial development of NMJs, eventually causing severe muscle weakness and premature death. In milder forms of SMA (types 2–4), the motor unit has completely developed, but SMN levels are too low to maintain structural and functional integrity of the motor unit. SMN levels are also too low to respond to nerve injury. Motor neuron loss and axonal degeneration eventually causes muscle weakness and atrophy

Unlike the inexorable progressive disease course of the motor neuron disease ALS, loss of muscle power (or simply failure to gain muscle strength) in SMA may be most evident at disease onset with subsequent stabilization for many years. This has led to the hypothesis that some aspects of SMA are due to impaired development of the motor unit. Histopathological analyses of spinal cords from patients with severe forms of SMA have shown both loss of anterior horn cells and signs of immature and mismigrated motor neurons. The number of large myelinated axons in ventral roots are reduced, but remaining axons show reduced diameters [48–51]. Neuromuscular junctions in SMA type 1 muscle show persistent expression of the fetal subunit of the acetylcholine receptor [52], and myofibers in patients with SMA type 0 and type 1 show ongoing apoptosis, as well as immature morphology [48, 51]. Together, these data suggest that while neuronal degeneration plays an important role, at least in patients with severe SMA, poor development of the motor unit may also occur (schematically shown in Fig. 3). Severe SMA mice appear to show a similar combination of both degenerative and developmental pathology. While there is motor neuron loss and denervation of a limited number of muscles, there is also impaired formation of mature, complex neuromuscular junctions, and retarded myofiber growth [40, 44, 47].

Delayed maturation of the motor unit suggests that there is a temporal requirement for SMN protein to develop a fully functional motor circuit. SMN expression is developmentally regulated in rodents and in humans. High expression is observed in gestational and early neonatal stages, followed by a sharp decrease to basal levels, which are maintained [52–55]. At the cellular level, SMN appears to be expressed at high levels in motor neurons at neonatal stages in rodents, nonhuman primates, and humans [52–55]. Transgenic mice that express SMN in an inducible, constitutive manner have indicated that restoration of SMN levels by early postnatal postsymptomatic stages is capable of substantially increasing survival [56, 57]. A recent study used a transgenic approach to ablate SMN expression in an inducible manner in mice, and confirmed that early SMN expression is required for proper development of the motor unit [58]. Interestingly, reducing SMN levels in adult mice had relatively little effect, further suggesting a temporal requirement for SMN [58]. Insensitivity to SMN deficiency emerged abruptly at postnatal day 17, which coincides with the time in development when mature neuromuscular junctions are established in mice [58]. The ability to regenerate mature neuromuscular junctions after injury in adult mice was impaired in the absence of SMN, suggesting that SMN might play a role in the maintenance of structural and functional integrity of the motor unit [58].

Together, these data provide evidence that SMN deficiency in early stages of development may cause neurodevelopmental abnormalities that may be relevant to patients with type 1 SMA (Fig. 3), while milder forms of SMA might be predominantly characterized by the inability to maintain integrity of motor units after their establishment. This could, in part, account for the differences in age of disease onset, disease severity, and survival between the types of SMA (Fig. 3).

## Therapeutic Strategies

Several neuroprotective drugs have been tested in SMA clinical trials. Riluzole is the only FDA-approved drug for the treatment of amyotrophic lateral sclerosis (ALS). Phase I clinical trials in patient with type I SMA showed that it was tolerated and that it showed similar pharmacokinetics in patients with SMA to those seen in patients with ALS [59, 60]. However, there was no evidence of efficacy, and these trials were complicated by patient drop out due to death, highlighting the challenges of clinical trials in this very fragile patient population.

Trophos developed a cholesterol-like compound (TRO19622, olexosime), which shows remarkable neuroprotective properties [61, 62]. Like riluzole, it was originally developed as a potential treatment for ALS [61, 62]. While its exact mode of action is not completely understood, it has been granted an orphan drug status for the treatment of ALS in the USA and for spinal muscular atrophy in Europe [62]. Recently, Trophos reported preliminary results at the 2014 Families of SMA meeting, indicating that olesoxime may prevent motor function loss in patients with type 2 and type 3 SMA, and other disease-related outcomes compared with the placebo-treated group over a period of 2 years in a phase II clinical trial (Fig. 2) [63]. Follow-up trials will indicate whether these preliminary results are clinically relevant.

Ever since the discovery of the unique genetics of SMA, SMN induction has been considered one of the most promising treatment strategies for the disease. Several approaches that indirectly induce SMN expression (e.g., the FDA-approved cyclo-oxygenase inhibitor celecoxib) [64] or that improve the SMA phenotype in an SMN-independent manner (e.g., the muscle growth pathway involving follistatin/myostatin) [65, 66, 67] have been tested in preclinical settings. However, most of them have been shown to have only limited effect in mouse models of SMA, might have side effects, or have low central nervous system (CNS) penetrance. However, several new drugs/biologics are entering clinical trials in patients, including those that aim to 1) increase *SMN2* expression; 2) modulate *SMN2* splicing; and 3) replace *SMN1* by gene therapy (Fig. 2). Some of these new therapeutic strategies are discussed below.

### *SMN2* Promoter Activation

**Fig. 4:**
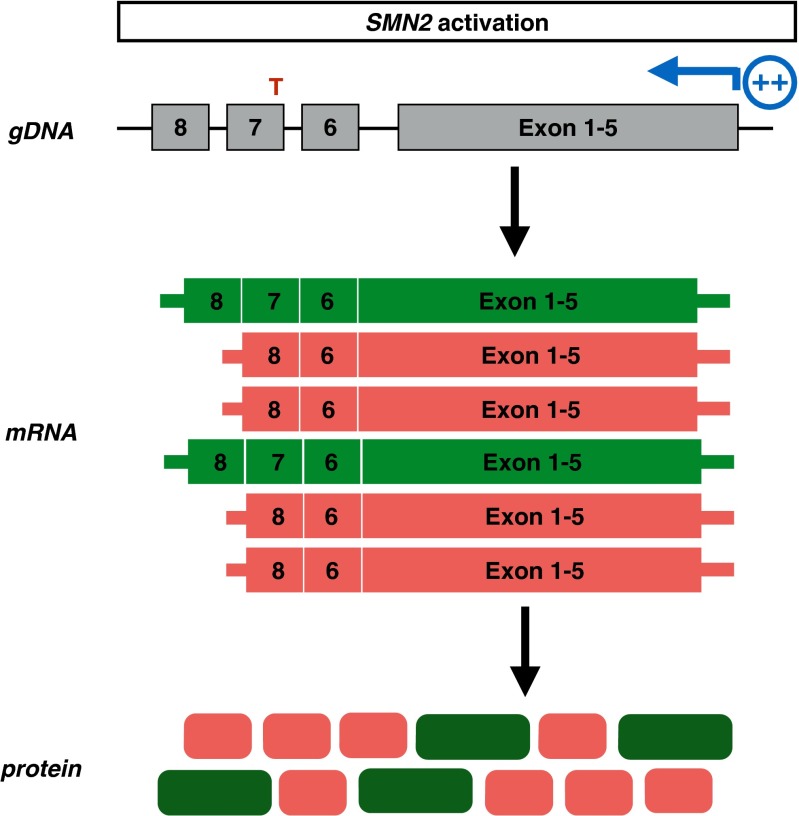
Mechanism of *SMN2* promoter activation drugs. *SMN2* promoter activating strategies aim to induce *SMN2* expression, resulting in increased full-length and truncated *SMN* mRNA and protein levels

Reduced SMN levels characterize SMA, and all patients with SMA retain at least 1 *SMN2* copy. An attractive potential therapeutic approach would be to increase the *SMN2* promoter activity (Fig. 4). Initial attempts to ameliorate the SMA phenotype were focused on increasing *SMN2* expression using histone deacetylase (HDAC) inhibitors. HDACs deacetylate histones thereby suppressing transcriptionally active genes. In 2001, Chang et al. [68] reported, for the first time, that the HDAC inhibitor sodium butyrate increased SMN levels in patient-derived cells and in mouse models of mild SMA. Since then, other reports have indicated that several HDAC inhibitors are able to increase SMN levels in patient-derived cells and in various mouse models of SMA. These HDAC inhibitors include valproic acid (VPA) [69, 70], the benzamide M344 [71], and the hydroxamic acid class of HDAC inhibitors, including suberoylnanilide hydroxamic acid [72–74], LBH589 [75], and trichostatin A [76, 77]. All HDAC inhibitors increased *SMN2* transcriptional activity, probably by their inhibitory effect on HDAC1 and/or HDAC2 [78]. However, these HDAC inhibitors had variable effects on SMN levels and variably improved the SMA phenotype in mice [73, 76, 77] perhaps owing, in part, to their low potency. Some of the HDAC inhibitors also exhibit low penetrance in the CNS. In addition, these compounds are pan-HDAC inhibitors, suggesting that they may have negative side effects. Despite their modest efficacy in preclinical models, HDAC inhibitors were evaluated in clinical trials in patients with SMA because 2 of them were already used in clinical practice for other indications. Clinical trials of VPA and phenylbutyrate indicated that HDAC inhibitors had little or no effect in patients with SMA type II and type III [79–81]. Patients treated for 1 year with VPA and L-carnitine showed excessive weight gain, affecting motor function in some cases [80]. Motor function appeared to be slightly improved when corrected for body weight in treated cases aged 2–3 years, but not in older cases [80]. These observations suggest that HDAC inhibitors might be beneficial when administered early [80]. In adult patients with type 3 SMA treated with VPA, only compound muscle action potentials modestly improved over the 1-year treatment period [81].

Besides acetylation/deacetylation, histones can also be methylated/demethylated, (de)phosphorylated, (de)ubiquinated and/or (de)sumoylated. In addition, DNA can be methylated at CpG islands, which also represses gene expression. All of those modifications have an impact on the chromatin structure, and hence on transcriptional activity [82–84]. For example, Hauke et al. [85] reported that *SMN2* is silenced by DNA methylation specifically at CpG islands within or in close proximity of the *SMN2* promoter. This observation suggests that targeting only 1 epigenetic marker of transcriptional activity might not be sufficient to increase expression levels of a given gene, or *SMN2* specifically. A systematic analysis of all these modifications might identify new promising therapeutic targets to increase *SMN2* promoter activity and consequently increase *SMN2* expression.

An alternative strategy to increase *SMN2* promoter activity was used by Jarecki et al. [86] in 2005 with support from “Families of SMA” [86]. In this study, an unbiased screen of 550,000 compounds was performed using a genetically engineered *SMN2* promoter assay in NSC34 cells, a motor neuron-like cell line. The most potent compounds that increased *SMN2* expression (both at the mRNA and the protein level) in this assay, as well as in patient-derived fibroblasts, were of the class of quinazolines [86]. In a follow-up study, the authors identified the scavenger mRNA decapping enzyme DcpS as the target of these compounds [87]. DcpS removes the residual 5’ cap after 3’–5’ degradation of mRNA. Now referred to as Repligen/Pfizer compound RG3039, this *SMN2* promoter activator only modestly increases *SMN* mRNA and SMN protein levels in various mouse models of severe and mild SMA [88–90]. Despite this modest increase, the DcpS inhibitor effectively extends the lifespan of SMA mice after oral administration [88–90]. Although the exact mechanism through which DcpS inhibition partially rescues the SMA phenotype is still under investigation, RG3039 is close to entering phase I clinical trials (Fig. 2).

### Modulation of Splicing

**Fig. 5:**
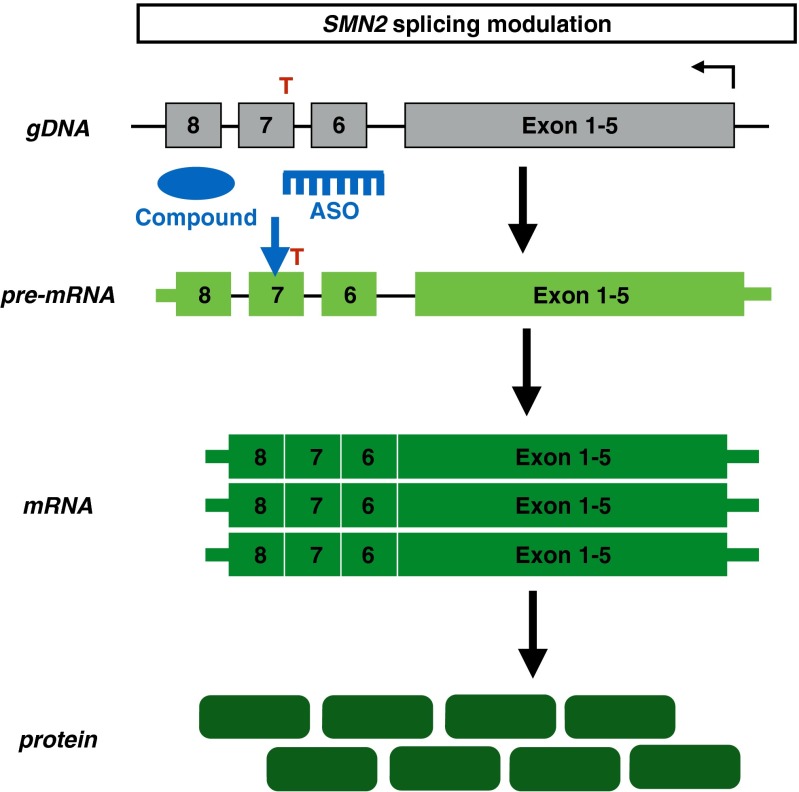
Mechanism of *SMN2* splicing modulation drugs. Antisense oligonucleotides (ASOs) or chemical compounds promote exon 7 retention in the *SMN2* pre-mRNA, resulting in increased full-length *SMN* mRNA and protein levels

As *SMN2* gene expression results in an alternative spliced, truncated, and nonfunctional product, an alternative approach for therapy development has focused on changing the splicing of *SMN2* pre-mRNAs (Fig. 5). Antisense oligonucleotide (ASO) technology was initially developed to downregulate gene expression by targeting mRNAs to induce their degradation or block their translation [91]. Advances in antisense chemistry now allow application of this technology to manipulate pre-mRNA splicing [91].

One approach to modulate splicing of exon 7 in *SMN2* was focused on shifting the competition between the 3’ splice site of exon 7 and exon 8 in favor of exon 7 inclusion by using antisense technology. Favoring exon 7 inclusion increased SMN protein levels dose-dependently in HeLa cells transfected with genetically engineered U7 snRNAs targeting a sequence partially overlapping the intron 7/exon 8 boundary (anti-*SMN* U7 snRNAs) [92].

Another approach to modulate exon 7 splicing aimed to block the intronic splice repressor Element1 (ISS-E1) sequence upstream of exon 7, as deletion of ISS-E1 increased exon 7 inclusion in HeLa cells [93, 94]. So-called bifunctional RNAs inhibit ISS-E1 and recruit SR proteins (conserved serine- and arginine-rich proteins involved in pre-mRNA splicing) to the SR recruitment sequence in the bifunctional antisense RNA to promote exon 7 inclusion [93]. These bifunctional RNAs increased exon 7 inclusion and SMN protein levels in transfected in HeLa cells and in patient-derived cells [93]. In addition, intracerebroventricular (ICV) delivery of bifunctional antisense RNAs in SMA mice variably increased SMN protein levels in the CNS system [93]. However, the phenotypic benefit of delivering bifunctional antisense RNAs in the CNS in SMA mice was limited [93]. Morpholino-based antisense treatment targeting Element1 by single ICV delivery elicited robust SMN protein induction in the CNS and muscle [95]. In addition, this treatment strategy improved survival, weight, motor function, and neuromuscular pathology in both severe and intermediate mouse models of SMA [95].

The identification of the intronic splicing silencer N1 (ISS-N1) element downstream of the 5’ splice site in intron 7 provided a third target for antisense technology to modulate *SMN2* splicing [96]. Deletion of ISS-N1 promoted exon 7 inclusion, indicating that ISS-N1 was another promising splice modulator target for *SMN2* [96]. Work from several laboratories has indicated that ASOs targeting ISS-N1 dramatically promoted exon 7 inclusion to near 100% of the primary transcripts, and increased SMN protein levels in transfected cell lines, in patient-derived cells, and in mouse models of SMA [96–100].

Treatment of adult heterozygous or wild-type transgenic *SMN2* mice twice a week (by tail vein injections) with this ASO resulted in a dose- and time-dependent increase in exon 7 inclusion in liver and kidney [98]. However, exon 7 inclusion rate was not affected in spinal cord of these animals as the ASO did not penetrate the blood–brain barrier (BBB) when injected systemically at this age [98]. An independent study using a different ASO targeting ISS-N1 (nucleotide range 13–33 downstream of exon 7) with a different backbone chemistry and delivered ICV to SMA mice increased SMN protein levels throughout the spinal cord and increased body weight and righting reflex [101]. In this study, the effect of the ASO on lifespan was not reported [101].

To investigate the effect of ASO in the spinal cord, Hua et al. [102] infused ASO 10–27 continuously for 7 days in the right lateral ventricle at various doses in adult mild SMA mice (type 3 SMA, *Smn* ^+/-^/*SMN2* ^+/+^ with 4 copies of *SMN2*). ASO treatment resulted in an almost complete rescue of exon 7 exclusion and increased SMN protein levels throughout all levels of the mouse spinal cord [102]. In addition, *in vivo* half-life of the ASO appeared to be very long, as the effect of the ASO was still observable 6 months after completing the 7-day treatment [102].

Alternatively, a single ICV administration of morpholino-based oligomers targeting ISS-N1 in neonatal Delta7 mice extended the lifespan to > 100 days [99, 100]. Delayed delivery of the morpholino to the CNS only had moderate efficacy with mild changes in lifespan, indicating that increasing SMN levels in early stages are critical to improve the SMA phenotype [99, 100].

Finally, the Krainer laboratory investigated the effectiveness of central *versus* systemic administration of ASO in the severe “Taiwanese” SMA mouse model [103]. In this study, ASO was administered intracerebroventricularly on day 2, or subcutaneously on day 1 or day 3 [103]. Delivery of the ASO in the CNS efficiently corrected *SMN2* splicing in brain and spinal cord, leading to increased SMN protein levels but only modestly extended survival (10 *vs* 16 days) [103]. In marked contrast, systemic delivery of the ASO resulted in a median survival of > 100 days [103]. Combining ICV and subcutaneous (SC) injections or 2 additional SC injections resulted in increased survival (173 and 137 days, respectively) [103]. Most rescued mice had nearly normal motor function, but ears and tails developed necrosis and were eventually lost [103]. ICV injection of the ASO resulted in a marked increase in exon 7 inclusion in brain and spinal cord, but had very limited effects in peripheral organs [103]. SC injections also increased exon 7 inclusion, including in brain and spinal cord, owing to the incomplete closure of the BBB in neonates and/or retrograde transport of the ASO [103]. The marked difference between ICV and SC injections on the rescue of the phenotype suggests that peripheral restoration of SMN expression is crucial for survival in this mouse model [103]. On a histological level, motor neuron counts, muscle fiber size, and neuromuscular junction integrity were similar between treated mice and their heterozygous controls [103]. Finally, the authors attributed the restoration of circulating insulin-like growth factor-1 secreted from the liver in treated SMA mice as a factor contributing to the rescue of the SMA phenotype [103]. These findings suggest that organs other than the CNS might contribute to SMA pathogenesis in mice. It is unclear if these findings are relevant to human disease, as the majority of patients with SMA do not have peripheral defects.

Based on the extraordinary effects of ASO 10-27 (now referred to as ISIS-SMNRx), ISIS Pharmaceuticals partnered with Biogen Idec and completed phase I (in 2013) and phase II (in 2014) clinical trials (Fig. 2). ISIS Pharmaceuticals/Biogen Idec reported that ISIS-SMNRx was well tolerated when administered intrathecally as a single dose. ISIS-SMNRx was also observed in the cerebral spinal fluid, indicating that the drug half-life in the human CNS is very long. In addition, single or multiple doses of ISIS-SMNRx also increased SMN protein levels and moderately increased the Hammersmith Functional Motor Scale-Expanded score, a functional read-out for motor function.

Given the complementary sequence of ASOs, ISIS-SMNRx very specifically modulates exon 7 splicing. Work in the mouse model has indicated that systemic delivery of the ASO has a superior effect on the SMA phenotype. Whether this would be the case in humans affected with SMA is thus far an unanswered question. While repeated intrathecal administration is an invasive route of delivery, the frequency of delivery is once every 3 months because of the long drug half-life.

In order to identify small molecules that might have similar effects on exon 7 splicing, there have been extensive screening efforts for chemical compounds that induce exon 7 inclusion. In 2001, aclarubicin was identified as a compound that increased exon 7 inclusion and SMN protein levels in in patient-derived fibroblasts [104]. Anthracycline antibiotics such as aclarubicin are widely used as part of conventional chemotherapy. However, their long-term use is prohibited owing to toxicity issues.

Based on the structural similarity to aclarubicin, Paratek Pharmaceuticals screened their tetracyclines derivatives library [105]. One derivative, PTK-SMA1, promoted exon 7 inclusion but did not change the splicing pattern of other genes, suggesting a specific effect on SMN2 exon 7 splicing [105]. PTK-SMA1 also increased exon 7 inclusion and SMN protein levels in a mouse model of mild SMA [105]. Paratek Pharmaceuticals is now in the final stages of optimizing PTK-SMA1 as an investigational new drug (Fig. 2). One challenge to this drug has been limited BBB penetration.

More recently, orally available compounds that modify SMN2 splicing in a quite specific manner have been identified by PTC Therapeutics and Roche in partnership with the SMA Foundation [106]. This study marks a major breakthrough in the search of compounds that specifically modulate a single splicing event. The compounds dose-dependently increased full-length *SMN* mRNA levels and SMN protein levels in patient-derived fibroblasts [106]. RNA sequencing indicated that these modifiers did not affect widespread gene expression changes as only 6 genes were up- or downregulated by a factor of 2 or more [106]. In addition, analysis of annotated splice junctions indicated that these compounds were highly specific in promoting SMN2 exon 7 inclusion [106]. These modifiers also increased motor function and dramatically extended survival of 2 different SMA mouse models when administered systemically by oral gavage and/or by intraperitoneal administration [106]. Independently, Novartis identified another orally available small molecule that is able to modulate splicing of *SMN2* and increase SMN protein levels in cell lines derived from SMA mouse models and patients with SMA [107]. Although the exact mechanism of action of these compounds is still not understood, clinical trials are being planned in Europe and the USA to evaluate the effectiveness of these orally available SMN2 splice modifiers (Fig. 2) [106].

### Gene Therapy

**Fig. 6:**
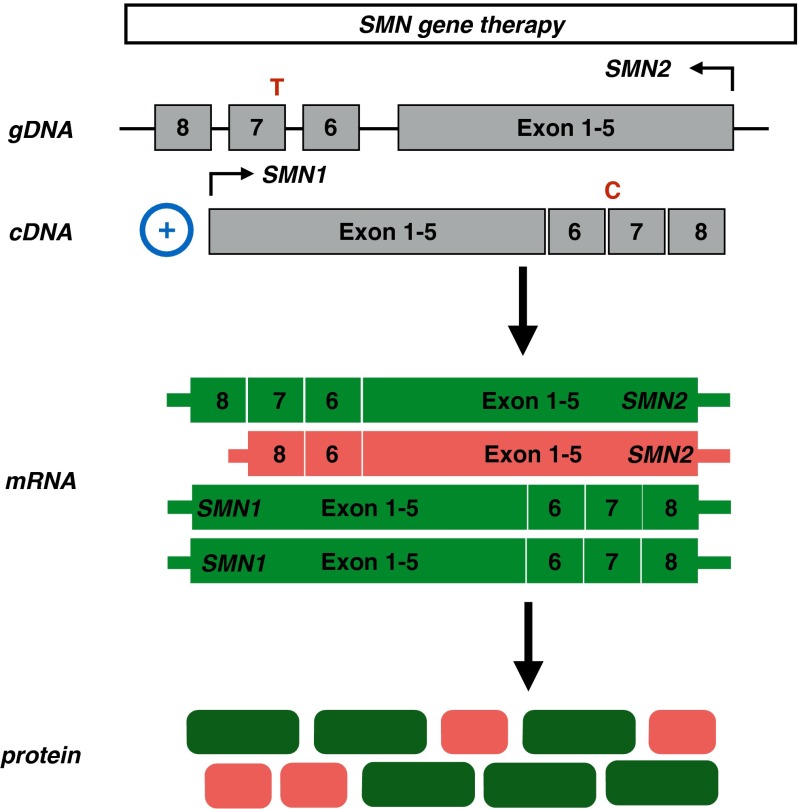
Mechanism of *SMN1* replacement by gene therapy. Using adenovirus-associated viruses, *SMN1* cDNA is incorporated in the human genome, resulting in increased full-length *SMN* mRNA and protein levels

Rather than increasing the expression of endogenous full-length *SMN2* by activating its promoter or by modulating splicing of exon 7, some groups have focused on replacing *SMN1* by gene therapy (Fig. 6). Ground-breaking work from the Kaspar laboratory [108] in 2010 showed that gene therapy had marked efficacy SMA mice. In this report, SMN cDNA under the control of a strong promoter was encapsulated by the self-complementary adeno-associated virus serotype-9 (scAAV9) [108]. The scAAV9 gene therapy delivered intravenously resulted in a marked increase in SMN protein levels in brain and in motor neurons of spinal cord, indicating that scAAV9 is able to traverse the BBB and has neurotropism. scAAV9 treatment also showed a striking increase in SMN protein levels in skeletal muscle [108]. One- or 2-day-old injected Delta-7 mice lived profoundly longer than the control mice (mean lifespan 250 *vs* 15.5 days, respectively) [108]. In addition, locomotor function, weight, and neuromuscular transmission were partially rescued [108]. Interestingly, when mice were injected at later time points, transduction with scAAV9 distributed more towards glial cells than motor neurons. Strikingly, lifespan of these treated animals dramatically decreased, suggesting that there is a very narrow therapeutic window in SMA mice [108]. The scAAV9 also proved to have clinical potential, as it traversed the BBB and efficiently infects cells in dorsal root ganglia and motor neurons within the whole spinal cord in a (1-day-old, injected) nonhuman primate [108]. Other work has demonstrated that transgene expression persists in the CNS (both motor neurons and glial cells in spinal cord), as well as skeletal muscles when nonhuman primates were systemically treated at various ages [109]. In addition, local scAAV9 injection into the cerebral spinal fluid of piglets efficiently transduced motor neurons throughout the spinal cord and resulted in robust transgene expression [109].

In other studies, AAV8–SMN1 has been studied in SMA mice. Injection of AAV8–SMN1 cDNA controlled by a promoter specific for the CNS resulted in increased weight gain, motor function, and a median survival benefit of 50 days [110]. However, the survival curve demonstrated a bimodal distribution, with a first group dying between 17 and 27 days, and the second group between 58 and 66 days [110]. One possibility is that the first group had a more severe SMA phenotype at the time of intervention. Alternatively, the first group might have had a more profound immune response against the AAV8 capsid compared with the second group [110]. The self-complementary version of this virus (scAAV8) showed more efficient targeting of choline acetyltransferase (ChAT) -positive cells in the spinal cord compared with the AAV8 version [110]. Treatment with scAAV8-SMN1 resulted in a striking improvement of median survival of 157 days [110]. Additionally, scAAV8-treated animals showed body weight gain, improved motor function, and remained ambulatory throughout their life. In both treatment strategies, animals developed hind limb necrosis albeit milder in the scAAV8-treated animals compared to the AAV8-treated mice [110]. AAV8-treated animals also displayed an increased number of choline acetyltransferase (ChAT)-positive cells compared with untreated animals, indicating that increasing SMN levels prevents motor neuron loss in the spinal cord. This results in the preservation of myofiber and neuromuscular junction architecture [110].

To determine if the route of injection significantly affects the degree to which the SMA phenotype is corrected, intravenous (IV) and ICV treatment was performed in 3-day-old SMA mice (with the same dose of scAAV9) [111]. Consistent with the original report, IV injection increased SMN protein levels in brain and spinal cord [111]. Brain SMN levels were increased dramatically in ICV-injected mice [111]. Both treatment strategies improved motor function of SMA mice [111]. Similar to the report of Passini *et al*. [110], a bimodal survival curve was observed in the IV-treated cohort, with some mice surviving only 25–35 days, while the remaining mice survived >200 days [111].

ICV or IV delivery of scAAV9 SMN in the severe SMN2 mouse model (with an average lifespan of 8 days) at day 1 (when the mice are already symptomatic) also results in weight gain (ICV better than IV) with robust SMN expression in brain and spinal cord [112]. IV-injected animals showed minimal improvement of the skeletal muscle architecture, while ICV displayed a slight improvement in muscle fiber area [112]. Nevertheless, motor function was not dramatically improved [112]. The extraordinary results with viral-mediated gene transfer in SMA mice and in nonhuman primates recently led AveXis to start a phase I clinical trial at Ohio State University (OSU) to assess safety and tolerability of intravenously injected scAAV9-SMN (referred to as “chariSMA”) in patients with SMA type 1 (Fig. 2). Patients who receive chariSMA must carry biallelic *SMN1* mutations and, at best, 2 copies of *SMN2*, and should have disease onset at birth or before the age of 6 months. These stringent inclusion criteria are, in part, to target patients as early in the disease course as possible. In addition, there are manufacturing limitations of the scAAV9 virus at this time that limit the size of an infant who can be treated systemically. Independently, Genzyme is also developing a viral-mediated gene therapeutic strategy to treat SMA (Fig. 2).

## Conclusions and Future Perspectives

As evident from this review (summarized in Fig. 2), there has been enormous progress in SMA therapeutics development in recent years. Several drug candidates have shown remarkable efficacy in mouse models of SMA, and we now stand on the verge of determining whether they will have efficacy in patients with SMA. Despite this optimism, there are still a number of questions that remain unanswered.

First, we need to address in what ways the SMA mouse models do or not recapitulate disease features of human SMA. The impairments of motor behavior and survival in SMA mice are likely relevant to human patients, but SMA mice at end-stage show significantly less motor neuron loss, as well as myofiber atrophy, compared with human patients. Whether patients with SMA have an early phase of disease characterized by functional abnormalities of motor neurons prior to degeneration similar to what is observed in mice and when this phase occurs is critical to address as this will be the optimum time for therapeutic intervention. Another issue is that many SMA mouse models develop ear, tail, and/or toe necrosis, and cardiac, gut, and urinary pathology that likely contribute to reduced survival in SMA mice, but may or may not be relevant in the human disease. These observations raise questions about whether cell types other than motor neurons show vulnerability to SMN deficiency in humans, and whether these contribute significantly to human SMA pathology.

Increasingly, it is recognized that preclinical testing of novel therapeutics in SMA mouse models needs to include assessments most relevant to patients, including motor neuron number, neuromuscular junction innervation, and muscle pathology, rather than simply survival. In addition, to overcome a bias based on mouse model-dependent outcomes, it is ideal to evaluate at least 2 SMA mouse models when investigating SMA pathogenesis and evaluating the effectiveness of new potential therapeutic strategies.

Second, it is clear from work done in SMA mice that restoring SMN levels genetically or pharmacologically early during development appears to be beneficial to extend survival, body weight, and motor function. Whether we can extrapolate this to patients with SMA is a matter of debate. An accurate profile of SMN protein expression both in controls and in affected individuals might shed light on a more accurate “window of opportunity” to induce *SMN* expression in humans. Information on how *SMN* expression in humans is regulated both at the transcriptional and post-transcriptional level is lacking. The fact that HDAC inhibitors only have a limited effect on *SMN* induction suggests that mechanisms other than repressive deacetylated histones might contribute to *SMN* silencing. A detailed investigation of these regulatory mechanisms might contribute to our insights on how SMN expression is temporally regulated in specific human cell types. Similarly, little is known about the splicing pattern of *SMN2* over time and/or in different human cell types. Mapping the frequency profile of exon 7 inclusion in *SMN2* might be useful when developing drugs that modulate SMN2 splicing.

Given the extraordinary rescue of the SMA phenotype in mouse models by some of the drugs, combining different strategies might have additive effects on SMN expression, and hence be more beneficial in treating SMA. For example, increasing SMN transcript levels using an *SMN2* promoter-activating drug in addition to a splicing modulator might increase SMN to levels higher than by treatment with only one of those drugs.

Finally, the genetics of SMA have led to the development of strategies to manipulate SMN expression directly. However, less extensive efforts have been devoted to identifying mechanisms that contribute to SMA pathogenesis either independently of SMN insufficiency or indirectly as a result of SMN insufficiency. An in-depth analysis of disease pathogenesis might identify novel disease modifiers (others than *SMN2*) as therapeutic targets

As the SMA drug pipeline has been growing and evolving rapidly over the last decade, and with dedicated researchers in the SMA field collaborating worldwide, we are hopeful that we stand, for the first time, within reach of a treatment for SMA.

## Electronic Supplementary Material

Below is the link to the electronic supplementary material.

ESM 1 (PDF 1225 kb)
